# Scanning Electron Microscope (SEM) Evaluation of the Interface between a Nanostructured Calcium-Incorporated Dental Implant Surface and the Human Bone

**DOI:** 10.3390/ma10121438

**Published:** 2017-12-17

**Authors:** Francesco Mangano, Mario Raspanti, Hassan Maghaireh, Carlo Mangano

**Affiliations:** 1 Department of Medicine and Surgery, University of Insubria, Varese 21100, Italy; mario.raspanti@uninsubria.it; 2 Dental School, University of Manchester, Manchester M139PL, UK; h.maghaireh@yahoo.co.uk; 3 Department of Dental Sciences, University Vita Salute S. Raffaele, Milan 20132, Italy; camangan@gmail.com

**Keywords:** scanning electron microscopy, nanostructured calcium-incorporated implant surface, human bone, osseointegration

## Abstract

*Purpose*. The aim of this scanning electron microscope (SEM) study was to investigate the interface between the bone and a novel nanostructured calcium-incorporated dental implant surface in humans. *Methods*. A dental implant (Anyridge^®^, Megagen Implant Co., Gyeongbuk, South Korea) with a nanostructured calcium-incorporated surface (Xpeed^®^, Megagen Implant Co., Gyeongbuk, South Korea), which had been placed a month earlier in a fully healed site of the posterior maxilla (#14) of a 48-year-old female patient, and which had been subjected to immediate functional loading, was removed after a traumatic injury. Despite the violent trauma that caused mobilization of the fixture, its surface appeared to be covered by a firmly attached, intact tissue; therefore, it was subjected to SEM examination. The implant surface of an unused nanostructured calcium-incorporated implant was also observed under SEM, as control. *Results*. The surface of the unused implant showed a highly-structured texture, carved by irregular, multi-scale hollows reminiscent of a fractal structure. It appeared perfectly clean and devoid of any contamination. The human specimen showed trabecular bone firmly anchored to the implant surface, bridging the screw threads and filling the spaces among them. *Conclusions*. Within the limits of this human histological report, the sample analyzed showed that the nanostructured calcium-incorporated surface was covered by new bone, one month after placement in the posterior maxilla, under an immediate functional loading protocol.

## 1. Introduction

For over thirty years, dental implants have been an effective solution for the restoration of the masticatory function, in the case of partially and completely edentulous patients [1,2,3,4]. The scientific literature has clearly demonstrated that dental implants are characterized by high survival and success rates, in the short [1,2] and long term [3,4].

The original protocol for the placement of dental implants was based on a submerged and undisturbed healing before functionalization, i.e., connection of the prosthetic abutment and placement of the provisional or definitive restoration [5,6]. This period, which comprised between four and six months, was considered necessary to obtain stable integration of the fixture into the bone, defined as osseointegration [5,6].

However, in recent years, patients have become more demanding and require early or immediate prosthetic restoration of the implants to avoid annoying periods with provisional removable prostheses [6,7,8]. Consequently, clinicians more and more often propose and use early [7,9] and immediate [8,10,11] loading protocols; this also takes place in areas characterized by poor bone quality (density) or quantity, and therefore is subjected to greater risk of failure [7,8,12]. 

Such variations of the classical surgical and prosthetic protocol “ad modum Branemark” [5,6] undoubtedly bring functional, cosmetic and psychological benefits to the patients (who do not need to wear uncomfortable removable prostheses or dentures during the healing period), as well as reducing the time and costs of treatment [7,8,9,10,11]. However, they pose a challenge, as the prosthetic load in the first healing period can result in micro-movements at the bone-implant interface, which beyond a certain threshold may lead to fibrous encapsulation of the fixture, and to the failure of osseointegration [12,13].

To meet the needs of patients and clinicians, a whole range of new micro-rough implant surfaces, produced by various subtractive [14,15] or additive [16,17] treatments, have been introduced on the market. Such treatments are intended to enhance and accelerate the phenomenon of osseointegration [14,15,16,17,18]. 

Histological and histomorphometrical studies in humans have shown that micro-rough surfaces can accelerate bone healing by stimulating the deposition of new bone on the fixture and thereby increasing the percentage of bone-to-implant contact, when compared to the conventional machined surfaces [14,16,19,20]. This may allow the clinician to anticipate the functionalization of the implant, even in difficult clinical contexts (situations with poor bone quality), and to propose and use early and immediate prosthetic loading protocols [7,8,9,10,11,12,18,19,20].

More recently, to further enhance and speed up bone healing mechanisms, dental implant companies have introduced new surfaces with a controlled nanophotography (i.e., nanostructured surfaces) [21,22,23,24]. In these implants, very often a controlled nanostructure is superimposed to a micro-rugosity [24]. 

Among these surfaces are those coated with nanoparticles and calcium ions [24,25,26]. The incorporation of calcium ions on the surface of a dental implant can in fact stimulate cell attachment, proliferation, alkaline phosphatase activity, and can upregulate gene expression of bone-related proteins [24,25,26]. 

Among the nanostructured calcium-incorporated surfaces are those obtained by discrete calcium-phosphate deposition (DCD) [27,28], those obtained by ion-beam assisted deposition (IBAD) of calcium ions [29,30,31], and finally those enriched with calcium ions through hydrothermal methods [24]. 

The Xpeed^®^ surface (Megagen, Seoul, South Korea) is a nanostructured surface enriched with calcium ions through a hydrothermal method [24,32,33]. Histological and histomorphometrical studies on humans have confirmed that this nanostructured surface can support new bone formation, under an immediate loading protocol [32,33].

However, there is not yet a literature study on the scanning electron microscope (SEM) evaluation of the interface between the human bone and this new nanostructured calcium-incorporated surface. 

Hence, the aim of this study is to investigate the interface between the bone and this new nanostructured calcium-incorporated surface in humans, through scanning electron microscopy.

## 2. Materials and Methods

### 2.1. Implant Surface Characteristics

The implant evaluated in the present study (Anyridge^®^, Megagen Implant Co., Gyeongbuk, South Korea) has a novel calcium-incorporated surface (Xpeed^®^). This nanostructured, calcium-incorporated surface is obtained as previously described [24], by modifying a surface produced by grit-blasting with particles of resorbable calcium phosphate (resorbable blast media, RBM), which is enriched with calcium using a hydrothermal method. In brief, RBM implants are immersed in a mixed solution of 0.2 M sodium-hydroxide (NaOH) and 2 mM calcium-oxide (CaO), dissolved in deionized water using a Teflon-lined hydrothermal reactor system at 180 °C for 24 h under a water pressure of 1 MPa^2^. With this procedure, a nanolayer of Ca^2+^ ions is incorporated onto the RBM surface, giving a CaTiO_3_ nanostructure. Previous studies [24,32,33] have reported the following standard roughness parameters for this surface: a mean Ra (the arithmetic mean of the absolute height of all points) of 1.6 (±0.2) µm, a mean Rq (the square root of the sum of the squared mean difference of all points) of 2.1 (±0.3) μm, and a mean Rt (the difference between the highest and lowest points) of 15.7 (±0.2) μm. 

### 2.2. Implant Placement and Timing of Loading

The fixture analyzed in this study (3.5 mm diameter × 10 mm length) had been placed in the posterior maxilla to replace a first premolar (#14) of a female patient who was 48 years old. The patient was a smoker (10 cigarettes/day) but with good oral hygiene. The implant was inserted with a conventional surgical protocol; that is, in a fully healed ridge (i.e., six months after the extraction of the compromised tooth). However, to meet the patient’s specific requests, the implant had been loaded immediately after placement with a single, temporary acrylic resin crown. The patient gave her informed consent for inclusion in the present study, which was conducted in accordance with the Declaration of Helsinki for medical research involving human subjects of 1975 (revision of 2008). The study protocol was approved by the Ethics Committee of Insubria University (project identification code #0034086, deliberative act 826).

### 2.3. Implant Retrieval and SEM Evaluation

After one month of functional loading, unfortunately, the patient was the victim of an accidental trauma. Following a road accident, she fell from the motorcycle and hit her face against the ground violently. The trauma caused fractures and mobilization of some natural teeth (#13 and #15), but it also affected the implant-supported restoration (#14): the fixture lost its stability and had therefore to be removed. Despite the violent trauma that caused the mobilization of the implant, immediately after removal, the surface of the fixture appeared to be covered by tissue. This tissue was firmly attached to the surface and appeared intact. Therefore, the fixture and the surrounding tissue were subjected to SEM examination. All procedures carried out in the present study were in full compliance with the principles described in the Declaration of Helsinki for medical research involving human subjects of 1975 (revision of 2008). The implant was fixed overnight in Karnowski, dehydrated with graded ethanol and hexamethyldisilazane, mounted on suitable stubs with conductive glue and gold-coated with an Emitech K550 sputter-coater (Emitech, Montigny Le Bretonneux, France). The specimen was then observed with a FEI XL-30 FEG high resolution SEM (FEI, Hillsborough, OR, USA) operated at an acceleration voltage of 7 kV using secondary electron imaging (SE). Pictures were directly obtained in digital format as 1424 × 968, 8 bpp TIFF grayscale files. Subsequently the same specimen was embedded in glycolmethacrylate (Technovit 7200 VLC, Heraeus Kulzer, Hanau, Germany), sectioned along its longitudinal axis with a high precision diamond-coated disk and polished flat. The specimen was then mounted as above, carbon coated with an Emitech K250 flash evaporator (Emitech, Montigny Le Bretonneux, France) and observed with the same FEG SEM operated at an acceleration voltage of 15 kV using backscattered electron (BSE) imaging. An unused AnyRidge^®^ screw was simply mounted on appropriate stubs with conductive glue without further treatments and observed as above, as a control.

## 3. Results

**Figure 1:**
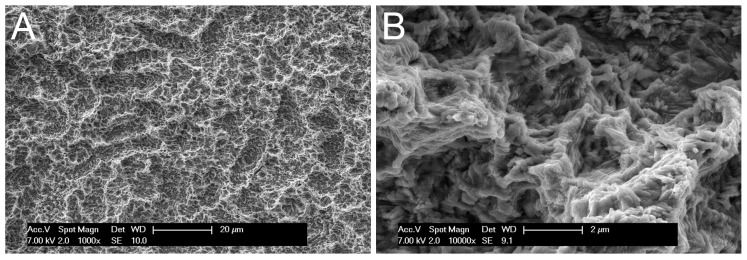
Scanning electron microscopy (SEM) pictures of the surface of an unused screw. (**A**) At low magnification (1000×), the surface is carved by irregular, multi-scale hollows reminiscent of a fractal structure; the specimen appears perfectly clean and devoid of any contamination. (**B**) At higher magnification (10,000×), picture shows the random topography and the fine texturing of the surface, limited only by the grain structure of the metal.

The surface of a new, unused screw showed a highly-structured texture, with nested nooks and cavities of a variety of sizes and orientations. Although not really fractal, the surface showed a sort of self-similarity with extends down to the grain structure of the metal (Figure 1A,B). The surface was clean and devoid of the spikes and blades which are typical of other techniques of surface texturing.

**Figure 2:**
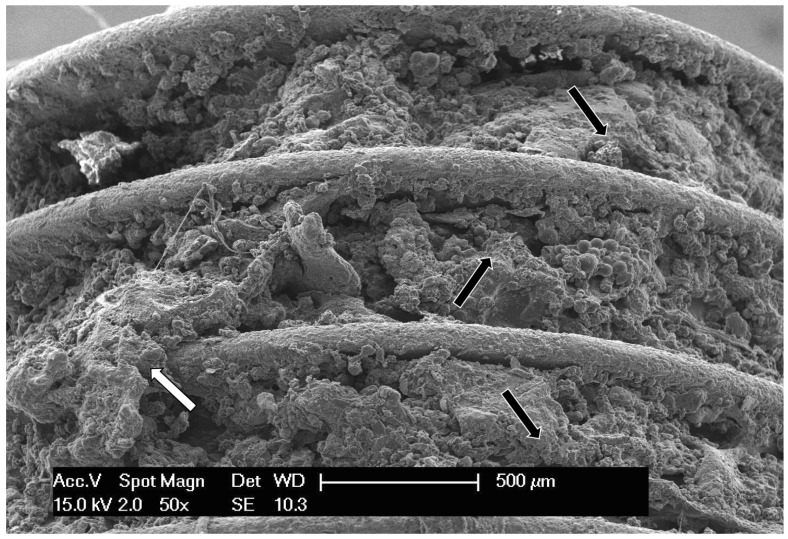
A low magnification (50×) electron micrograph of the implant. Three ridges of the screw are visible across the image, left to right. The voids among the threads are entirely occupied by growing bone tissue (black arrows): the new bone covering the entire fixture confirms the early osseointegration process. On the left, a bone patch crosses the metal ridges (white arrow).

**Figure 3:**
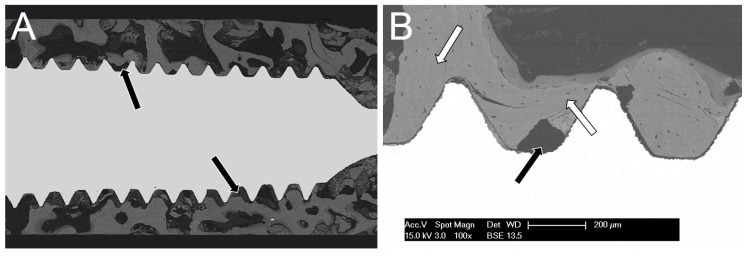
(**A**) A mosaic of several backscattered electron micrographs shows the entire mid-section of the implant and reveals how the implant surface is almost entirely covered by newly formed bone. With this technique, the titanium screw appears as solid white and the bone as gray while soft, unmineralized tissues are not apparent. The dense trabecular structure of the bone is readily evident. (**B**) A detail (100×) of the screw-tissue interface. Different shades of grey represent different phases of bone deposition, with older bone appearing brighter (i.e., more mineralized) (white arrows) than neo-deposed bone (black arrows). The narrow gap separating the screw and the tissue is an artifact due to the different shrinkage rates of the two components.

The lateral surface of the implant (Figure 2), as well as its resin-embedded midsection (Figure 3A), confirmed that the entire surface of the screw was involved in the osseointegration process. The implant was entirely surrounded by trabecular bone anchored to the metal surface, which bridged the screw threads and filled the peri-implantar space; the presence at the interface of several tissues, from bony trabeculae to small clusters of adypocytes, indicated a relative maturity of the integration process even after such a short period (one month). At higher magnification (Figure 3B), the early osseointegration process was again confirmed by the presence of new bone covering the entire fixture. In particular, the different phases of bone deposition and maturation were clearly evident, with older, more mineralized bone appearing brighter than newly deposed bone.

## 4. Discussion

Bone is a natural nanostructured material (i.e., a material with constituent features less than 100 nm in at least one dimension), made of organic compounds (mainly collagen) reinforced with inorganic compounds (hydroxyapatite). It is this natural nanostructure that nanotechnology aims to emulate for dental applications [20,21,22,34,35]. Since titanium appears to be more tied to bone when a thin ceramic layer is grown on its surface, through chemical or thermal treatments, several nanostructured surfaces for calcium ion incorporation have been introduced in the market [21,22,23,24,27,28,29,30,31].

Among the nanostructured, calcium-incorporated surfaces, there are surfaces enriched with calcium ions by a hydrothermal method, like Xpeed^®^ [24,32,33].

Although several clinical studies have shown that dental implants with a nanostructured surface enriched with calcium ions through the hydrothermal method (Xpeed^®^) can have high survival and success rates, at least in the short term [10,36,37,38,39], still little is known about the mechanism of healing these fixtures in vivo [32,33].

The best way to evaluate osseointegration in vivo is to retrieve an osseointegrated implant from the human native bone and to analyze the bone-implant interface histologically and istomorphometrically [14,16,19,21]. Obviously, this type of evaluation can be difficult for ethical reasons, and often temporary, mini-implants that are easier to retrieve are used in these studies [14,16,19,21,32,33]. 

Not surprisingly, very few human histological studies are present in the scientific literature with reference to other types of nanostructured calcium-incorporated surfaces, such as those obtained from discrete calcium-phosphate deposition (DCD) [40,41,42]; such studies were based on very few samples, and the evidence that emerged from them was limited. Moreover, to our knowledge, no human histological and histomorphometrical studies are available on nanostructured surfaces obtained through ion-beam assisted deposition (IBAD) of calcium ions. For these surfaces, the only evidence in the literature derives from animal histological studies [29,30,31,43,44].

The surface enriched with calcium ions through the hydrothermal method has been studied in more detail, and recently two human histological and histomorphometrical studies have evaluated the early bone response to nanostructured, calcium-incorporated implants [32,33].

In a randomized controlled histological/histomorphometrical study in the human posterior maxilla, the authors compared the early (eight weeks) bone response following the insertion of implants with a nanostructured calcium-incorporated surface (Xpeed^®^) with that determined by the insertion of implants with a machined surface [32]. In this study, 15 fully edentulous patients received two temporary transmucosal fixtures, according to a split-mouth design. One had a nanostructured, calcium-incorporated surface (*test*) and the other a machined surface (*control*) [32]. All implants were immediately loaded, to help to support an interim complete maxillary denture. After a healing period of eight weeks, all temporary transmucosal implants were removed with a trephine drill, for histological/histomorphometrical evaluation. Bone-to-implant contact (BIC%) and bone density (BD%) in the threaded area were evaluated, and the results obtained with *test* and *control* implants were compared [32]. In the machined implants (*control* group), the histomorphometrical evaluation revealed a mean BIC% (±SD) and BD% (±SD) of 21.2% (±4.9) and 29.8% (±7.8), respectively [32]. In the nanostructured, calcium-incorporated implants (*test* group), the histomorphometrical analysis revealed a mean BIC% (±SD) and BD% (±SD) of 39.7% (±8.7) and 34.6% (±7.2), respectively [32]. A statistically significant difference was found between the two surfaces with regard to BIC% (*p* < 0.001) [32]. Therefore, the authors concluded that the incorporation of calcium ions onto the implant surface through the hydrothermal method seems to increase the peri-implant endosseous healing properties in the native bone of the posterior maxilla, under immediate loading conditions [32].

In another human histological and histomorphometrical study [33], the authors investigated the effects of fixture design and surface on the early osseointegration of immediately loaded implants in the posterior maxilla. In this study, 10 fully edentulous patients received two transmucosal transitional fixtures, according to a split-mouth design; one was a conical implant with a knife-edge thread design and a nanostructured calcium-incorporated surface (*test*) and one a cylindrical implant with a self-tapping thread design and a sandblasted surface (*control*) [33]. All implants were immediately loaded for a period of two months, and they were therefore retrieved for histological/histomorphometrical examination [33]. Once again, the BIC% and BD% in the threaded area were evaluated, and the results obtained with *test* and *control* implants were compared [33]. With *test* implants, a mean BIC% and BD% of 35.9 (±9.1) and 31.8 (±7.5) were reported, whereas with *control* implants, a mean BIC% and BD% of 29.9 (±7.6) and 32.5 (±3.9) were seen [33]. Although the bone contact was higher with the nanostructured, calcium-incorporated surface, no statistically significant difference (*p* = 0.16) was found in the BIC% between *test* and *control* implants [33]. Finally, similar BD%s were found in the two groups (*p* = 0.9) [33]. The authors concluded that under immediate loading conditions, the knife-edge thread design and the nanostructured calcium-incorporated surface can somehow help to increase bone formation and accelerate healing processes, when compared to cylindrical fixtures with a sandblasted surface [33]. 

Our present SEM report seems to confirm the evidence emerging from these studies. In fact, one month after the placement and immediate loading in the posterior maxilla, the surface of the nanostructured calcium-incorporated fixture was covered by newly formed bone tissue, capable of filling the spaces between the threads and even creating “bridges” between them. The osseointegration process was relatively mature even after a short period (one month), with the different phases of bone maturation in evidence. 

It is important to emphasize that this result was achieved in the posterior maxilla one month after implant placement, under the immediate functional loading of a standard-sized fixture. The posterior maxilla is generally characterized by a rather poor bone quality compared to other anatomic sites, and is therefore one of the most dangerous sites in the positioning of dental implants [6,8,9,12,13,18]. Furthermore, in our study, the implant was subjected to immediate functional loading. Immediate loading represents an additional risk factor for implant failure in the short term, especially in the posterior maxilla, and in the case of single, non-splinted implants [6,8,9,12,13,18]. In fact, the scientific literature supports the concept that micro-movements transmitted by occlusal forces and oral tissues (lips, cheeks, tongue) to the bone-implant interface, above a certain threshold, may jeopardize osseointegration, and determine the mobilization and failure of the implant [5,6,7,8,9,12,13]. Finally, in our present SEM study, we report on the results obtained with an implant of standard dimensions (3.5 mm diameter × 10 mm length), and not with transitional nor temporary implants of reduced dimensions, like in previous studies [16,19]. This may represent an additional advantage of our study, because we have evaluated how the healing processes take place in a real situation.

Our present study has limitations. First, it is only a case report, and logically it would be advisable to evaluate a larger number of samples to draw more specific conclusions about the effectiveness of this nanostructured calcium-incorporated surface in accelerating and stimulating the deposition of new bone in the early healing phases. Moreover, the fact that the implant was removed after a trauma might have somehow affected our SEM assessment, and it would have been preferable to intentionally remove the fixture (and not as a result of a motorcycle accident), to better evaluate an untouched bone-implant interface. For all these reasons, further SEM studies on a larger sample of patients and with a different design (i.e., intentional removal of a series of fixtures) are needed to confirm the positive outcomes emerging from our present report.
